# SILAC-Based Quantitative Proteomic Analysis of Human Lung Cell Response to Copper Oxide Nanoparticles

**DOI:** 10.1371/journal.pone.0114390

**Published:** 2014-12-03

**Authors:** Mariola J. Edelmann, Leslie A. Shack, Caitlin D. Naske, Keisha B. Walters, Bindu Nanduri

**Affiliations:** 1 Institute for Genomics, Biocomputing and Biotechnology, Mississippi State University, Mississippi, United States of America; 2 Department of Basic Sciences, College of Veterinary Medicine, 240 Wise Center Drive, Mississippi State University, Mississippi, United States of America; 3 Department of Chemical Engineering, Mississippi State University, Mississippi, United States of America; Banaras Hindu University, India

## Abstract

Copper (II) oxide (CuO) nanoparticles (NP) are widely used in industry and medicine. In our study we evaluated the response of BEAS-2B human lung cells to CuO NP, using Stable isotope labeling by amino acids in cell culture (SILAC)-based proteomics and phosphoproteomics. Pathway modeling of the protein differential expression showed that CuO NP affect proteins relevant in cellular function and maintenance, protein synthesis, cell death and survival, cell cycle and cell morphology. Some of the signaling pathways represented by BEAS-2B proteins responsive to the NP included mTOR signaling, protein ubiquitination pathway, actin cytoskeleton signaling and epithelial adherens junction signaling. Follow-up experiments showed that CuO NP altered actin cytoskeleton, protein phosphorylation and protein ubiquitination level.

## Introduction

Copper (II) oxide (CuO) nanoparticles (NP) have widespread applications in industry, such as paint, heat transfer fluids, and semiconductors. CuO NP have applications in medicine, including antimicrobial materials [1]–[3] to treat *Tinea pedis* fungal infection [4], protection against human influenza virus H1N1 [5], and might also have applications in cancer treatment, due to its ability to induce apoptosis in cancer cells [6]. Engineered CuO NP can be released into the environment and have negative impacts on human health. Indeed, CuO NP have neurotoxic effects [7], such as alteration of dopamine system-related gene expression and enhanced dopamine depletion [8], as well as negative effects on voltage-dependent potassium currents in pyramidal neurons [9]. The CuO NP cytotoxic effects are dose-dependent [10]–[13], and size-dependent, with nanoparticles being more toxic than micrometer particles of the same metal oxide [14], [15], which is likely due to the damage that CuO NP cause in mitochondria. NP of other metal oxides, such as SiO_2_ and Fe_2_O_3_, have been shown to be non-toxic in the same experimental setting [11]. Comparison of CuO NP to TiO_2_, ZnO, CuZnFe_2_O_4_, Fe_3_O_4_ and Fe_2_O_3_ NP also demonstrated that CuO NP was relatively more cytotoxic and induced cell death and DNA damage [9]. However, it is known that these negative cellular impacts are not due to exposure to Cu ions alone as *in vitro* exposure to Cu ions in solution did not induce the same intracellular reactive oxygen species (ROS) formation, oxidative DNA damage and cell death that is seen in corresponding CuO NP exposure studies [12], [16], [17].

Recently, a DNA microarray study was done in A549 lung epithelial cells exposed to CuO NP. Epithelial cell exposure to NP is expected to represent the response of lung barrier function during inhalation exposure, a common route of NP exposure [18]. Although the effects on cell cycle arrest and generation of ROS was shared between Cu ions released from the NP and CuO NP, CuO NP affected additional processes such as nucleobase, nucleoside, nucleotide and nucleic acid metabolic processes. Very limited information is available regarding the response of cells to CuO NP at the protein level. A gel-based proteomics approach of murine macrophages identified forty-six differentially expressed proteins in response to CuO NP and eight proteins differentially expressed in response to Cu ions, of which five proteins were common to both treatments [19]. Analysis of these proteins showed that Cu ions altered expression of proteins involved in general stress response, while functions more specific to macrophages such as phagocytosis could be attributed to CuO NP alone. These studies were useful in the identification of cell death mechanisms triggered by CuO NP. However, the proteome coverage reported in the proteomics study is limited. To date, global quantitative proteomics methods have not been applied to study the effects of CuO NP exposure on mammalian cells. For inhalation exposure, which is one of the common routes of particle exposure in humans, epithelial cells are an appropriate choice for assessing nanoparticle cytotoxicity. Therefore, we chose human epithelial cells to study the effect of CuO NP on the proteome. In our study, first we evaluated the response of BEAS-2B human lung cell proteome to CuO NP, using SILAC-based mass spectrometry. Secondly, since phosphorylation is one of the most abundant protein post-translational modifications regulating key molecular processes, and based on our initial proteomics results showing it was expected to be altered, we also did quantitative analysis of CuO NP-modulated phosphorylated peptides using SILAC proteomics. Expression level of several key proteins was altered upon CuO NP exposure including proteins relevant in cellular function and maintenance, protein synthesis, cell death and survival, cell cycle and cell morphology. We also detected significant changes in signaling pathways such as mTOR signaling, protein ubiquitination pathway, actin cytoskeleton signaling and epithelial adherens junction signaling.

## Materials and Methods

### Characterization of CuO NP

The copper (II) oxide (CuO) nanoparticles (NP) used in this study were acquired from Alfa Aesar (CAS 1317-38-0, MW 79.55, PN 44663). The size reported by the vendor was 30–50 nm, with the following properties: 13 m^2^/g (surface area), 6.3–6.49 g/cm^3^ (density), 1,326°C (melting point), and 2.63 (refractive index). To resuspend the CuO NP in growth medium, Dulbecco's-modified Eagle's medium (DMEM)/F12 medium (Invitrogen) supplemented with 10% FBS and 1% penicillin/streptomycin (#15140-122, Invitrogen) was used. To prepare CuO NP in water, we used ultrapure water (18.2 MΩ•cm at 25°C) from a Millipore Synergy UV Type 1 water filtration system.

### Dynamic Light Scattering (DLS)

DLS particle size analysis was performed in phosphate buffered saline (PBS, pH 7.4), water and methanol. For PBS, a Mobius SR11-132 (Wyatt Technology Corporation) with a 170 µL sample volume was utilized at a CuO NP concentration of 1 mg/mL. CuO NP samples were also analyzed in water and methanol using a Malvern Instruments Nano-zeta dynamic light scattering system at a more dilute concentration of 0.00025 mg/mL. The equilibrium time between measurements was 2 min and the total measurement time for each sample was 60 sec.

### Electron Microscopy

Scanning electron microscopy (SEM) images were collected using a JEOL JSM-6500F field emission scanning electron microscope (FE-SEM). The CuO NP powder was pressed onto carbon tape. Transmission electron microscopy (TEM) characterization was performed with a JEM-2100 transmission electron microscope using Gatan US4000 and EMAN2 e2boxer.py. The pixel size was established as 1.919 nm/pixel for images recorded at 6,000X magnification at 80 kV. An Oxford EDS system was used for elemental analysis and mapping. Solutions of the CuO NP were prepared in purified water and cell culture growth media at a target concentration of 0.08836 µg/mL, to mimic cell culture conditions. Just prior to deposition, each solution was sonicated for 5 min and vortexed for 1 min. Solution droplets (1–3 µL) were placed on carbon-nickel TEM grids; the grids were maintained at 37°C for up to 24 h (until the samples were dry). Particle size analysis was performed using ImageJ software (version 1.44, released January 31, 2011). Images were scaled using the scale bar, the threshold was set to include all particles and the wand tracing tool was used to select and measure a minimum of 200 particles or all particles for smaller sample sets. Average values are reported along with 95% confidence intervals.

### Cell culture and CuO NP treatment

The BEAS-2B cells were purchased from the American Type Culture Collection (ATCC) and cultured exactly as described by others [20] in DMEM/F12 medium (Invitrogen) supplemented with 10% FBS and 1% penicillin/streptomycin (#15140-122, Invitrogen). The cells were grown in tissue culture dishes at 37°C in a 5% CO_2_ incubator. Cells were plated on tissue culture dishes and left for 24 h to attach and stabilize before the addition of CuO NP.

A stock CuO NP suspension (1 mg/mL) was prepared using PBS and it was diluted to an appropriate concentration using the DMEM/F12 culture media. Before adding to the cells, CuO NP were dispersed for 5 min by using a bath sonicator (Cole-Palmer) to prevent aggregation and then vortexed for 1 min. The nanoparticles were then added to the cells and the medium was gently swirled several times to ensure distribution of the CuO NP on the plates.

### Toxicity assay

**Figure 4:**
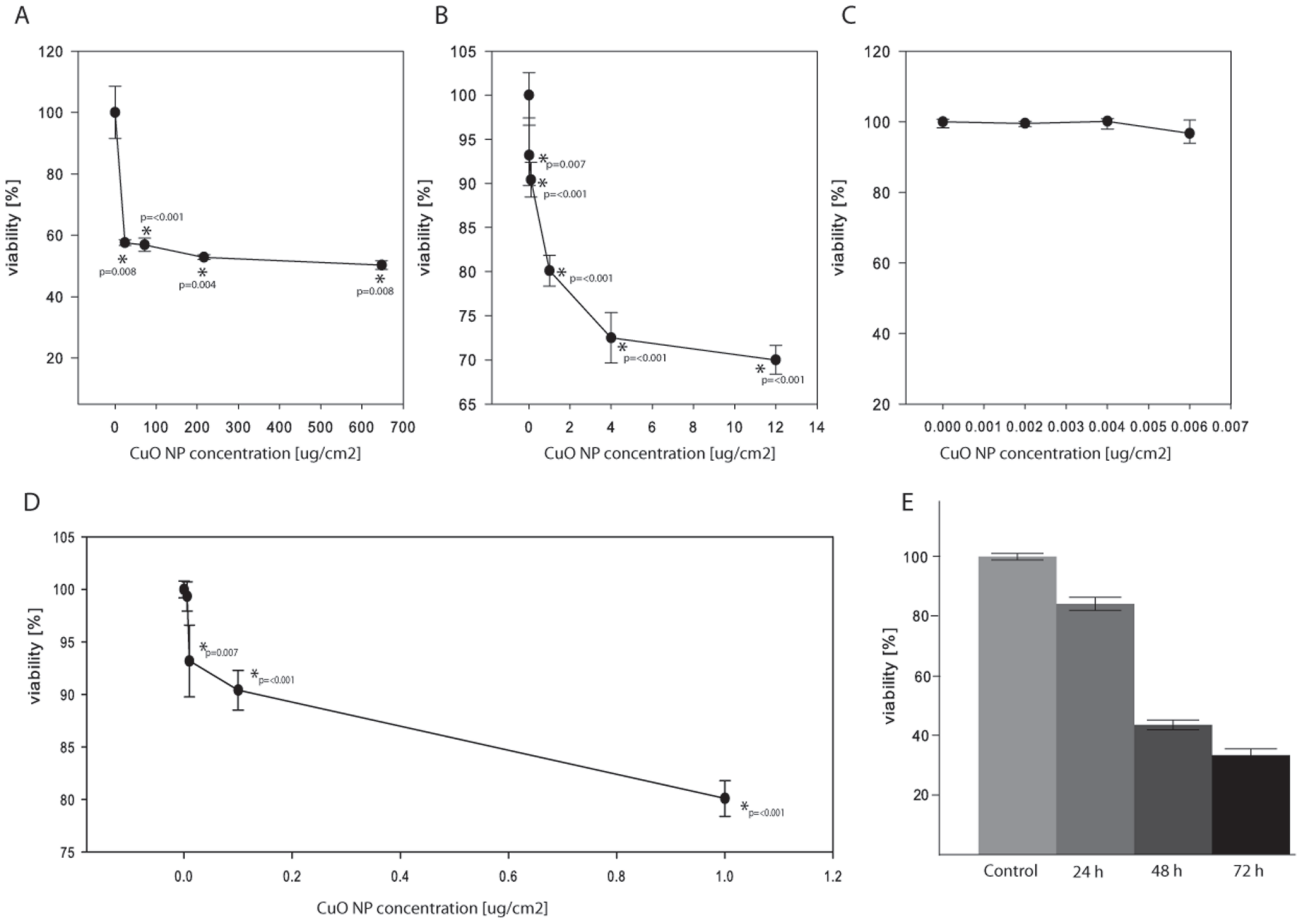
The CuO NP affects viability of human BEAS-2B lung cells. (A–D) BEAS-2B cells were plated on 96-well plates, left to recover for one day and then exposed to the CuO NP (30–50 nm) for 24 h at concentrations displayed on the graph. The cell viability was measured by the Alamar Blue assay and the statistical analysis (T-test) was done using SigmaPlot. The p values are displayed for the concentrations with statistically significant changes. CuO NP concentrations are displayed at different scales for readability. (E) BEAS-2B cells were plated on 96-well plates, left to recover for one day and then exposed to the 0.01 µg/cm^2^ CuO NP (30–50 nm) for 0 h, 24 h, 48 h and 72 h. Cell viability was measured by the Presto Blue assay.

BEAS-2B cell viability at different CuO NP concentrations was measured by the Alamar Blue assay (Invitrogen). Cells were seeded on 96-well special optics low fluorescence Assay Plates (Corning) with 6.5×10^3^ in 100 µL media per well. After a 24 h stabilization of the cells, 100 µL of the CuO NP (concentrations used were as indicated in Fig. 4) suspended in DMEM/F12 cell media was added to the cells. Cells were exposed to the CuO NP for 24 h. At the end of exposure, 10 µL of the Alamar Blue reagent was added to each well and the cells were incubated for an additional 2 h at 37°C. The fluorescence was then quantified using the fluorescence microplate reader on the qPCR system (Stratagene Mx3005P) and ROX/Texas Red filter (585 nm–610 nm). The viability of the treated group was calculated as a percentage of non-treated control cells, which was assumed to be 100%. T-tests were conducted using SigmaPlot (12.1) to identify significant changes in cell viability (p-value of ≤0.05) in response to CuO NP administration.

Cell viability at different time points and at a single concentration of CuO NP (0.01 µg/cm^2^) was measured by the Presto Blue assay (Invitrogen). 6.5×10^3^ BEAS-2B cells in 100 µL media per well were seeded on 96-well tissue culture plates. After a 24 h stabilization, CuO NP suspended in DMEM/F12 cell media (at 0.01 µg/cm^2^ concentration corresponding to 0.090 µg/mL) was added to the cells. Cells were exposed to the nanoparticles for 0 h, 24 h, 48 h and 72 h. At the end of exposure, 10 µL of the Presto Blue reagent was added to each well and the cells were incubated for 2 h at 37°C. The fluorescence was then measured by a SpectraMax M5 (Molecular Devices) fluorescence microplate reader where the monochromator was set to 560 nm excitation and 590 nm emission. The viability of the treated group was calculated as a percentage of non-treated control cells, which was assumed to be 100%. A cell viability graph was prepared by using JMP Pro 11.0.0 software.

### SILAC cell culture for proteomics

The BEAS-2B cells were cultured in DMEM/F12 (1∶1) media for SILAC without L-Lysine and L-Arginine (#88215, Thermo Fisher) supplemented with dialyzed Fetal Bovine Serum (#89986, Thermo Fisher) and either ‘heavy’ amino acids (^13^C_6_ L-Arginine-HCl and ^13^C_6_ L-Lysine-2HCl, Thermo Fisher) or ‘light’ amino acids (^12^C_6_ L-Arginine-HCl and ^12^C_6_ L-Lysine-2HCl, Thermo Fisher) and 1% streptomycin/penicillin (#15140-122, Invitrogen). The cells were routinely split by using Cell Dissociation Buffer (#13151-014, Invitrogen). After approximately five doublings, the incorporation of ‘heavy’ and ‘light’ amino acids into the cells was tested using mass spectrometry as described [21] using SILAC Protein Quantitation Kit - DMEM:F12 (#88439, Thermo Fisher), following manufacturer's instructions. Cells were prepared for the CuO NP treatment, exactly as described in the ‘Cell culture and the CuO NP treatment’ section. Approximately 10×10^6^ BEAS-2B cells from the ‘heavy’ cell population were treated with 0.01 µg/cm^2^ CuO NP, while the ‘light’ cell population was left as a control. The experiment was also repeated in reverse. The cells were then lysed in 0.1% NP-40 lysis buffer (0.1% NP-40, 150 mM NaCl, 20 mM CaCl_2_, 50 mM Tris pH 7.4) containing protease inhibitor cocktail (Roche Applied Science) and phosphatase inhibitor cocktail (PhosSTOP, Roche). The protein concentration was determined using the Bio-Rad Protein Assay Kit II (Bio-Rad) and equal amounts of protein from ‘light’ and ‘heavy’ populations were mixed together in a 1∶1 ratio which is common to SILAC-based proteomics approaches to ensure uniformity in the downstream sample preparation processes, thereby reducing errors and improving quantitation by mass spectrometry.

### Mass Spectrometry

Equal amounts of protein from BEAS-2B cells exposed to CuO NP for 24 h and grown in light and heavy amino acids were subjected to chloroform/methanol precipitation and digested with trypsin in-solution, exactly as we did before [22], [23]. After digestion, the samples were dried in a vacuum centrifuge and subjected to purification by SepPak C18 columns (Waters), as we described earlier [22], [23]. Some of the peptide samples were subjected to phospho-enrichment using immobilized-metal affinity chromatography (IMAC) or analyzed directly by LC-MS/MS. The tryptic peptides were solubilized in 50 µl of water containing 2% acetonitrile and 0.1% formic acid and separated by using a 75-µm I.D. ×15 cm reverse phase column (fused-silica C18 column, Thermo Fisher) controlled by an Ultimate 3000 nanoflow HPLC (Dionex). The elution of peptides was done using a 180 min gradient from 2%–95% solvent B (95% acetonitrile, 0.1% formic acid) at a flow rate of 0.5 µL/min. Peptides were then introduced into an OrbiTrap Velos mass spectrometer (Thermo Fisher), which was operated in data-dependent mode, automatically switching between MS and MS/MS. Full scan MS spectra (300–2,000 amu) were acquired with a resolution of 60,000 and analyzed by an FT-MS analyzer. The five most intense ions were selected for collision-induced fragmentation in the OrbiTrap at normalized collision energy of 35% and activation time of 10 min.

Mass spectra and tandem mass spectra were analyzed by Proteome Discoverer 1.3 (Thermo Fisher) using SEQUEST algorithm and a Uniprot human protein database (#3A181, downloaded on 01.23.2013) and a reversed human decoy database. Searches were done using a precursor tolerance of 10 ppm and a fragment mass tolerance of 0.9 Da. The following dynamic modifications were included: N-terminal acetylation; oxidation on methionine; carbamidomethylation on cysteine; 13C(6)15N(4) modification on arginine (+10.008 Da); and 13C(6) modification on lysine (+6.020 Da). Also, phosphorylation on serine, threonine and tyrosine was included for the datasets where phosphopeptides were enriched. The results were filtered using normalized XCorr values for different charge states [24], and were accepted as valid identifications only if the precursor ion mass accuracy was below 10 ppm and the XCorr values were >1.5, >2 and >2.25 for singly, doubly and triply charged peptides, respectively, with a minimum Delta Cn of 0.1.

For quantification of the SILAC data (Heavy/Light ratio calculation), the built-in SILAC 2-plex quantification method was used (Proteome Discoverer 1.3, Thermo Fisher) using Arg-10 and Lys-6 labels, where single-peak quant channels were allowed. To account for the experimental bias, the normalization was done by using the intensities of twenty proteins. Data for the untreated and CuO NP-treated cells labeled with ‘heavy’ and ‘light’ amino acids were combined for identifying significant changes in protein expression. Proteins with opposing trends in expression in these two independent experiments (i.e. biological replicates) as well as the proteins that were identified with less than 1.5 fold change in expression were discarded from further analysis.

### Ingenuity Pathway Analysis

In order to identify significant molecular functions, networks and signaling pathways represented by the total proteins as well as the differentially expressed (DE) proteins identified by SILAC, we used the Ingenuity Pathway Analysis software (IPA; Ingenuity Systems Inc) exactly as we have done earlier [25]. Using the human genome as a reference, IPA performed a one-tailed Fisher's exact test to determine whether the overlap of the molecular functions and pathways represented in our datasets was significant and generated a p value. A p value of ≤0.05 was considered to be significant. The p value for molecular functions and canonical pathways was corrected for multiple testing by the Benjamini-Hochberg method to ensure statistical stringency. The top ten functions and pathways were chosen for interpretation of the underlying biology. From the list of differentially expressed proteins, IPA also generated networks of no more than 35 molecules. Networks identified with a score of ≤2 were considered to be significant. This score represents the probability that the molecules in a network were identified to be interacting by random chance. We used IPA to retrieve the functional annotations and pathways represented by phosphorylated proteins.

### Immunoblotting

Approximately 5×10^6^ BEAS-2B cells treated with 0.01 µg/cm^2^ CuO NP for 24 h were lysed in 0.1% NP-40 lysis buffer (0.1% NP-40, 150 mM NaCl, 20 mM CaCl_2_, 50 mM Tris pH 7.4) containing protease inhibitor cocktail (Roche Applied Science) and phosphatase inhibitor cocktail (PhosSTOP, Roche). 4x reducing sodium dodecyl sulfate (SDS) buffer (62 mM Tris, 10% glycerol, 4% sodium dodecyl sulfate, bromophenol blue, 15% β-mercaptoethanol, pH 6.8) was added to the protein samples, which were then denatured for 3 min at 95°C and resolved by SDS polyacrylamide gel electrophoresis (SDS-PAGE). The proteins were transferred onto a polyvinylidene fluoride (PVDF; Millipore) membrane for 45 min at 95 V in blotting transfer buffer (25 mM Tris, 190 mM glycine, 20% methanol). Membranes were blocked in PBS with 5% milk and 0.05% Tween-20 for 1 h, washed in PBS containing 0.05% Tween-20, then probed with an appropriate primary antibody diluted in PBS containing 1% milk and 0.05% Tween-20 at 4°C overnight. After washing four times in 0.05% Tween-20 solution in PBS, horseradish peroxidase (HRP)-conjugated secondary antibody at the appropriate dilution in 1% milk, 0.05% Tween-20 in PBS was added to the blots. The ECL+ (Amersham) reagent was used for visualization and the blots were exposed to film (Kodak, Sigma-Aldrich).

The following antibodies were used for immunoblotting: antiphosphotyrosine antibody (1∶2000 dilution; 4G10 Platinum, #05-1050, Millipore), anti-beta actin antibody (1∶4000 dilution; #A1978, Sigma-Aldrich), anti-HA antibody (1∶10000 dilution; #H9658-.2 ML Sigma-Aldrich), anti-ubiquitin (1∶8000 dilution; #550944, BD Pharmingen), peroxidase (HRP)-conjugated anti-mouse antibody raised in goat (1∶3000 dilution; #P044701-2, DAKO). To visualize protein loading, the membranes were stained with Ponceau S Red stain (Acros) and destained with 5% acetic acid.

### Phosphorylation enrichment by IMAC

Phosphopeptide enrichment was carried out by the Immobilized Metal Affinity Column (IMAC) method using PHOS-select gallium silica spin columns (Sigma-Aldrich). After tryptic digestion of 500 µg protein sample, the peptides were subjected to purification using C18 SepPak columns (Waters), lyophilized in a vacuum centrifuge and reconstituted in 50 µL Bind/Wash solution containing 250 mM acetic acid and 30% acetonitrile. The PHOS-select columns were washed twice by addition of 50 µL of Bind/Wash solution and centrifugation for 30 sec at 500×g. The peptide sample was then loaded onto the PHOS-select columns and incubated for 15 min at room temperature, followed by centrifugation for 30 sec at 500×g. The flow-through was collected and re-loaded onto the PHOS-select columns in the same manner. The columns were then washed five times with 50 µL of Bind/Wash solution and once with 50 µL of Millipore H_2_O. The elution of phosphopeptides was done by addition of 50 µL of 400 mM ammonium hydroxide, vortexing and centrifugation for 30 sec at 500×g. The elution was repeated twice and the eluate was collected, lyophilized in a vacuum centrifuge and resuspended in 0.1% formic acid, 2% acetonitrile for LC-MS/MS analysis.

### Labeling of protein lysates with ubiquitin-specific active-site probes

BEAS-2B cells were exposed to the CuO NP at 0.01 µg/cm^2^ and 0.02 µg/cm^2^ for 24 hr. The cells were lysed and equal amounts of protein were incubated with ubiquitin-specific active-site probes (ubiquitin vinyl methyl ester (Ub-VME) and ubiquitin bromoaldehyde (Ub-Br); Enzo Life Sciences) for 45 min at 37°C, or left without any treatment, followed by SDS-PAGE and anti-HA immunoblotting, which was used to visualize active deubiquitinases that reacted with the HA-tagged probes.

### Actin cytoskeleton staining

In order to analyze changes in actin cytoskeleton structure in BEAS-2B cells treated with CuO NP, cells were cultured on coverslips and exposed to 0.01 µg/cm2 CuO NP for 24 h at 37°C. Cells were then washed for 30 sec at 4°C in wash buffer (Cytoskeleton Inc.), fixed for 10 min in 4% paraformaldehyde, washed three times for 30 sec at 4°C in wash buffer. Cells were permeabilized for 5 min with permeabilization buffer, and washed for 30 sec at 4°C in wash buffer, and stained for 30 min with Rhodamine Phalloidin by using the F-actin Visualization Biochem Kit (Cytoskeleton Inc.) following manufacturer's instructions. The cells were then covered with mounting medium containing DAPI (Vectashield). The images were collected by using Confocal Laser Scanning Microscopy (Zeiss Axiovert 200 M Inverted Research Microscope) and processed by using LSM Image Browser (4.2.0.121). Images from at least four replicates were collected for each condition.

## Results and Discussion

### CuO NP characterization

Nanoparticle sizes and the corresponding volume and surface area are critical variables to be considered when examining the impacts of nanoparticle exposure. Reported nanoparticle dimensions are often those measured immediately after synthesis. These dimensions can change dramatically during agglomeration or dissolution over time, during experimental manipulations or as a result of the methods used during characterization (e.g. agglomeration and precipitation induced by solution evaporation). Particle size and particle size distributions (PSD) of the CuO NP used in this study were assessed in both the dry ‘as received’ state and also under *in situ* cell culture conditions.

**Figure 1:**
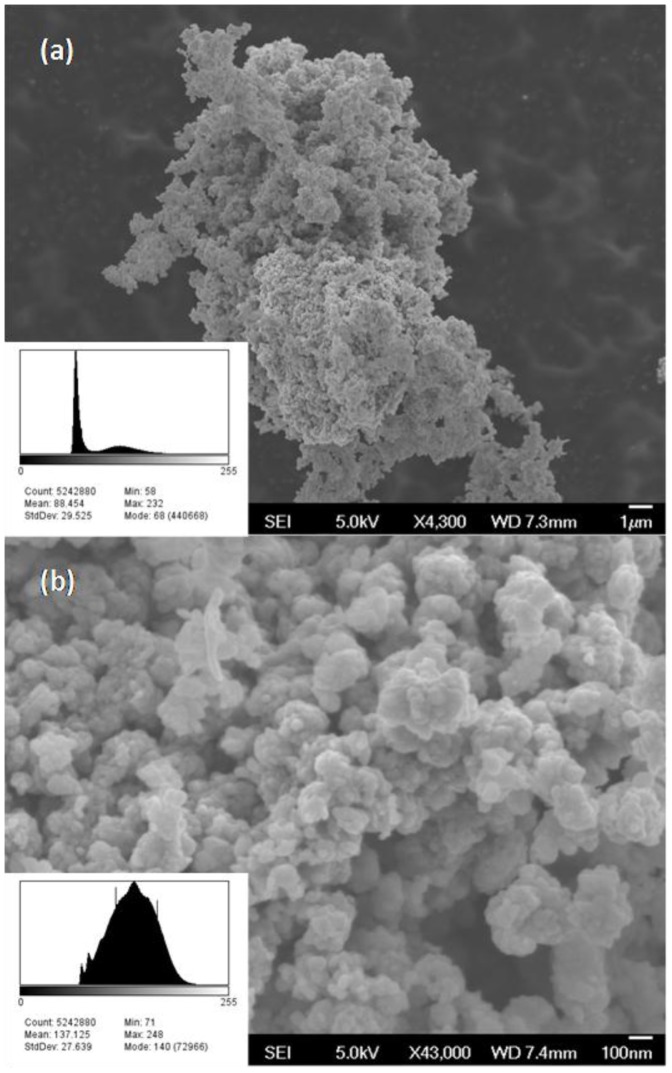
Scanning electron microscopy images of CuO NP showing the agglomerated powder particle structure. Particle size measurements were made from scanning electron microscopy (SEM) images of the CuO powder in its ‘as received’ state.

Particle size measurements were made from scanning electron microscopy (SEM) images of the CuO powder in its ‘as received’ state. These measurements consistently showed average particle diameters between 60 and 250 nm, with an average particle diameter of 130 nm (Fig. 1), and are much larger than the dimensions reported by the vendor. The CuO NP were then examined in-solution using dynamic light scattering (DLS). The hydrodynamic diameter of CuO NP in PBS at room temperature was found to be 224+/−22 nm. The zeta potential and mean mobility were measured at −0.057 mV and −2.97 (µm cm)/(V sec), respectively. DLS measurements in water and methanol resulted in larger average hydrodynamic diameters of 272.63+/−3.63 nm and 317.30+/−17.54 nm, respectively. These particle size measurements correlate with the order of solubility parameters: PBS>H_2_O>methanol. Also the particle-particle and particle-fluid interactions are likely to be governed primarily by the polar and hydrogen-bonding parameters [19]. The *in situ* particle size results were larger than expected, and so additional particle size investigations were pursued.

**Figure 2:**
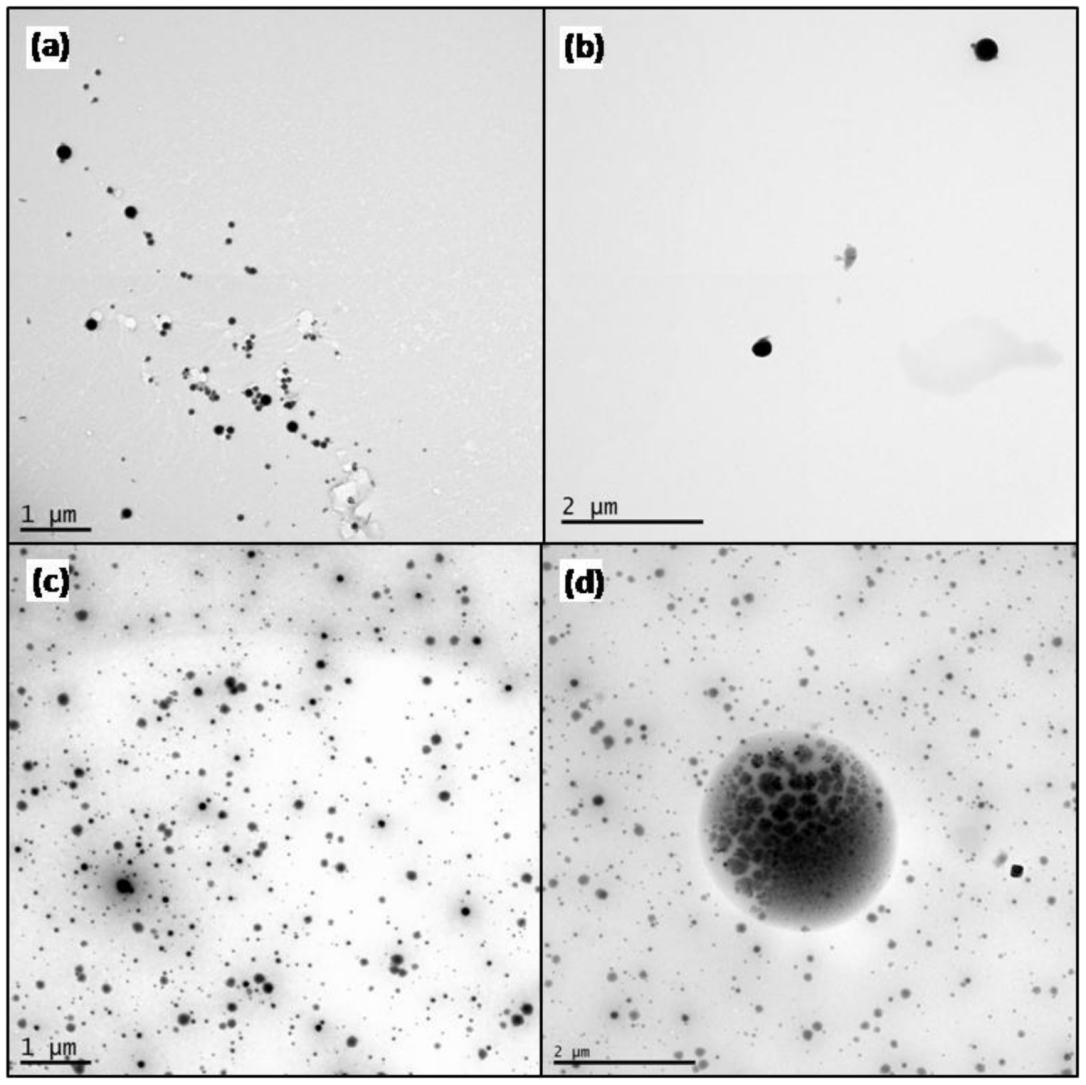
Transmission Electron micrographs of CuO NP deposited from water (A–B) and growth media (C–D). Number, size, and density of the particle phase were significantly altered by the solvent despite identical mass loadings of the CuO NP in solution.

Transmission electron microscopy (TEM) images were collected on CuO NP deposited from either purified water or growth media after incubation at the same incubation temperature (37°C) and time (24 h) as was used for the cell culture study (Fig. 2). The goal was to gather images showing primary, secondary (small cluster) and perhaps tertiary (porous, floc-like structures made up of the small clusters) particle structures. Although CuO NP concentrations were the same in both sets of solutions, there was a significant difference in the particle number, size and density of the particulate.

TEM was carried out in water (Fig. 2 A–B) and under *in situ* cell culture conditions (Fig. 2 C–D). An average particle size of 56.2+/−2.9 nm was measured for the growth media solution which is close to the vendor-reported size (Table 1). In contrast, global proteomics study with RAW264 cell one and CuO NP reported a particle coated with PVP was close to 250 nm in RPMI medium [19]. As expected, there was variation in the particle size in water sample that showed a larger average particle diameter of 85.6+/−7.2 nm. Agglomeration is apparent in both the growth media and water solution samples, but agglomeration is more extensive in the water solution as demonstrated by a maximum agglomerate diameter of ∼685 nm (65% larger than the maximum size measured in the growth media samples). The circularity of agglomerates in the water sample points to aggregation of the CuO NP in the water in contrast to the more diffuse behavior in the growth media.

**Table 1:** CuO NP particulate size distributions deposited from growth media and aqueous solutions measured by TEM.

	Diameter (nm)							
	Avg	STDEV	95% CI	Min	Max	Circularity	Aspect Ratio	Roundness
**Growth Media** * **(n = 694)**	56.2	38.3	2.85	2.04	234	0.55+/−0.01	1.2+/−0.02	0.85+/−0.01
**Water (n = 238)**	85.6	56.6	7.22	13.3	386	0.76+/−0.02	1.22+/−0.03	0.84+/−0.02

* measurements exclude the large agglomerate shown in Fig. 2D that was determined to be a statistical outlier.

**Figure 3:**
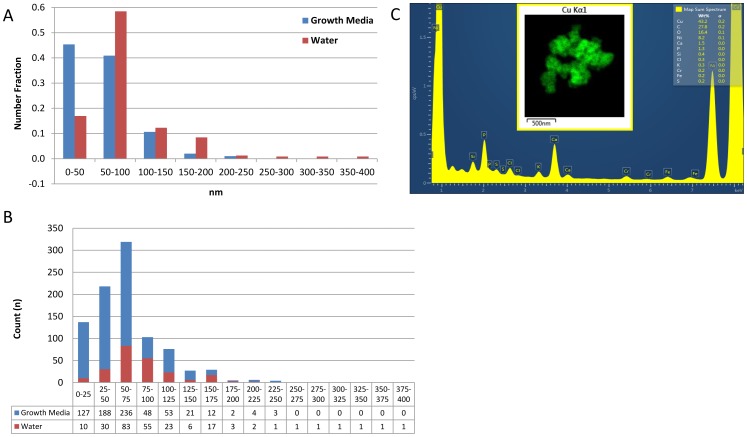
TEM images of CuO NP agglomerates and particle size counts from growth media and water. Number of CuO NP and NP agglomerates from sample prepared from growth media (blue) and water (red) for transmission electron microscopy, and particle size counts for CuO deposited from growth media and water (A–B). CuO NP more readily agglomerated in water, but both solutions showed the presence of CuO nanostructures ranging from <50 nm to > 200 nm in diameter. The increased agglomeration in water resulted in a smaller number of larger sized CuO nanostructures in solution. The energy-dispersive X-ray spectroscopy spectrum and Cu Kα1 map for a CuO NP cluster deposited from growth media is shown (C).

While CuO NP agglomerated more readily in water, both solutions showed the presence of CuO nanostructures with diameters ranging from <50 nm to >200 nm. Initial CuO NP concentrations in the growth media and water solutions were the same. Total particle volume detected by TEM was similar for the two solutions (a 14.85% differential), demonstrating that the results obtained are highly representative of the entire sample. The CuO NP were still in solution after the 24 h incubation at 37°C. Therefore, an increase in particle/agglomerate size would correspond to a lower particle count. This was observed in the water sample TEM image (Fig. 2 A–B), which had an average measured diameter of 85.6 nm, but approximately a third the number of particles (Table 1). Particle count reduction can also be observed in Fig. 3 A–B where the growth media had a higher count of 0–50 nm particles and water had larger particles in the range of 250–400 nm.

The solubility of CuO in water is limited but has been found to increase with temperature and phosphate concentration [26]. So the results seen here are not unexpected. However, additional studies are needed to determine the relationships between the ambient conditions of the cells of interest including salt species, ionic strength, pH and temperature along with the resultant CuO NP particle and aggregate sizes and solubilization of the metals. The chemical identity of the particulates was confirmed with energy-dispersive X-ray spectroscopy (EDS, Fig. 3C). After removing the C and Ni content (from the Formvar grid and adventitious C sources), the primary components were Cu (67.5 wt%) and O (25.625 wt%). Evaluation of speciation will be an important part of future efforts.

The environmental conditions—both global and local to the cells—are key to deducing toxicity correlations. In addition, the valence of the metal species is important [18], [27], [28]. TEM image collection was performed over two days with the same set of samples. During this time, the outer ‘shell’ of the NP became less dense than the core and indicated a partial dissolution of the CuO NP as they were left in the growth media. This phenomenon was not observed for CuO NP, at least not to the same degree, in the purified water solution. The metal valence, size and concentration are tied to the cell environment and all these will impact the toxicity. Transmission electron microscopy appears to mimic the *in situ* growth conditions used in the present study for proteomic analysis. An average particle size of 56.2+/− 2.9 nm reported by TEM is the possible size of the majority of CuO NP that was used to determine changes in protein expression in cells. However, TEM characterization did indicate that although the predominant proportion of CuO NP in growth medium was in the 50 nm range, the cells were indeed exposed to a wide range of particle sizes. Since the known biological effects of NP are size-dependent, future studies where the NP are size-fractionated (barring agglomeration) are warranted.

### CuO NP cytotoxicity

The CuO NP has been shown to have a toxic effect on various human cell types [12]. We used human epithelial lung cell line, BEAS-2B, which has been previously used in other studies concentrating on nanoparticle toxicity [20]. We determined BEAS-2B cell viability at different concentrations of the CuO NP after 24 h exposure with this nanoparticle by using the Alamar Blue assay. The results (Fig. 4 A–D) indicate that at a concentration of 8 µg/cm^2^ CuO NP had a significant effect on cell viability (Fig. 4A). A marginal yet statistically significant effect on cell viability (7%, Fig. 4B, D) was observed with 0.01 µg/cm^2^ CuO NP. However, CuO NP does not cause a significant change in BEAS-2B cell viability at a concentration range of 0–0.006 µg/cm^2^, as the viability remains at >97% (Fig. 4C, D). Based on these results, we chose to study the proteome response at 0.01 µg/cm^2^ CuO NP to identify cellular response mechanisms not overshadowed by the known effects on apoptotic mechanisms that NP elicit in cells. To confirm that 24 h is the optimal time point for further experimentation, we determined BEAS-2B cell viability using a complementary Presto Blue assay, which is very similar to the Alamar Blue assay. Cells were exposed to 0.01 µg/cm^2^ CuO NP for 0 h, 24 h, 48 h and 72 h. Even with the Presto Blue assay we saw that 24 h exposure registered the smallest decrease in cell viability at 85% (and 91% for the upper 95% mean, relative to the control), as compared to the 72 h treatment, where the viability was just 33% relative to the control (Fig. 4E).

The selected CuO NP dosage for experimentation by SILAC proteomics is ∼2.5 log orders lower when compared to the published microarray study that identified cell death mechanisms in the A549 cell line [18]. In the microarray study, effects of equivalent concentrations of released Cu ions and CuO NP (25 µg/mL CuO NP) on gene expression were studied and indicated that Cu ions affected less than one tenth of the genes that were differentially expressed in response to CuO NP. Proteomics study using CuO NP and Cu ions in mouse RAW 264.7 cells clearly demonstrated that certain cellular processes affected are specific to CuO NP and that Cu ions elicit response at a much smaller scale than the corresponding CuO NP concentration used for treatment of cells [19]. The concentration of CuO NP for analyzing the BEAS-2B proteome reported here is one log lower than the proteomics study [19], i.e. the equivalent Cu ion concentration will also be log orders lower, thus it is not expected to cause any measurable response in protein expression. Therefore, we did not conduct proteomics with released Cu ions and instead chose to conduct validation of some of our proteomics findings with CuO NP. The rate of internalization of CuO NP by BEAS-2B cells could also be important in explaining some of these effects, and could be explored further in future studies.

### SILAC Proteomics and Pathway analysis

SILAC based mass spectrometry identified a cumulative total of 186 proteins from the untreated control and CuO NP (24 h) treated cells (Tables S1–S3 in Information S1). Of these, expression of 84 proteins was found to be significantly different between the control and treatment datasets (Table S4 in Information S1). Expression of 48 proteins increased while 36 proteins showed decreased expression upon exposure to CuO NP (Table 2).

**Table 2:** BEAS-2B proteins with significant changes in expression upon CuO NP exposure for 24 h.

ID	Entrez Gene Name	Symbol	Fold Change
O60488	Acyl-CoA synthetase long-chain family member 4	ACSL4	−97.4
H7BZ94	Prolyl 4-hydroxylase, beta polypeptide	P4HB	−88.8
P61204	ADP-ribosylation factor 3	ARF3	−88.3
P30084	Enoyl CoA hydratase, short chain, 1, mitochondrial	ECHS1	−64.7
Q04760	Glyoxalase I	GLO1	−60.8
O75531	Barrier to autointegration factor 1	BANF1	−49.5
H7C4J1	ARP3 actin-related protein 3 homolog C	ACTR3C	−45.2
I3L192	Basigin (Ok blood group)	BSG	−37.1
P38646	Heat shock 70kDa protein 9 (mortalin)	HSPA9	−12.1
I3L3Q7	Complement component 1, q subcomponent binding protein	C1QBP	−9.9
B8ZZ54	Heat shock 10kDa protein 1 (chaperonin 10)	HSPE1	−8.2
E7EQR4	Ezrin	EZR	−6.6
H0YDT6	Eukaryotic translation initiation factor 3, subunit F	EIF3F	−6.2
F5H7E2	Superkiller viralicidic activity 2-like 2	SKIV2L2	−6
P14866	Heterogeneous nuclear ribonucleoprotein L	HNRNPL	−4.6
P13667	Protein disulfide isomerase family A, member 4	PDIA4	−4.6
P62081	Ribosomal protein S7	RPS7	−4.3
H0YGW7	ATP-binding cassette, sub-family F (GCN20), member 1	ABCF1	−3.5
F8W7F7	Transmembrane emp24 protein	TMED4	−3.4
E9PBF6	Lamin B1	LMNB1	−3.2
Q09666	AHNAK nucleoprotein	AHNAK	−3
P04075	Aldolase A, fructose-bisphosphate	ALDOA	−2.6
Q01518	CAP, adenylate cyclase-associated protein 1	CAP1	−2.5
B4DUR8	Chaperonin containing TCP1, subunit 3 (gamma)	CCT3	−2.3
P09972	Aldolase C, fructose-bisphosphate	ALDOC	−2.2
F8VVM2	Solute carrier family 25, member 3	SLC25A3	−2.2
H7BZJ3	Protein disulfide isomerase family A, member 3	PDIA3	−2
Q9BUF5	Tubulin, beta 6 class V	TUBB6	−2
P23526	Adenosylhomocysteinase	AHCY	−1.9
E9PLD0	RAB1B, member RAS oncogene family	RAB1B	−1.8
P60953	Cell division cycle 42 (GTP binding protein, 25kDa)	CDC42	−1.7
B7Z254	Protein disulfide isomerase family A, member 6	PDIA6	−1.7
P07737	Profilin 1	PFN1	−1.7
K7EJB9	Calreticulin	CALR	−1.6
C9JYS8	Non-POU domain containing, octamer-binding	NONO	−1.6
P14314	Protein kinase C substrate 80K-H	PRKCSH	−1.5
H0Y7A7	Calmodulin 1 (phosphorylase kinase, delta)	CALM1	1.6
P37802	Transgelin 2	TAGLN2	1.6
C9J5V9	Y box binding protein 1	YBX1	1.6
B4DQH4	Chaperonin containing TCP1, subunit 8 (theta)	CCT8	1.7
P11142	Heat shock 70kDa protein 8	HSPA8	1.9
P68036	Ubiquitin-conjugating enzyme E2 L3	UBE2L3	1.9
B4DW05	Prohibitin 2	PHB2	2
P46782	Ribosomal protein S5	RPS5	2
O43707	Actinin, alpha 4	ACTN4	2.1
A8K8G0	Hepatoma-derived growth factor	HDGF	2.1
P32119	Peroxiredoxin 2	PRDX2	2.1
Q9NYL9	Tropomodulin 3 (ubiquitous)	TMOD3	2.2
B4DLW8	DEAD (Asp-Glu-Ala-Asp) box helicase 5	DDX5	2.3
P24534	Eukaryotic translation elongation factor 1 beta 2	EEF1B2	2.4
P30041	Peroxiredoxin 6	PRDX6	2.6
G3V1A4	Cofilin 1 (non-muscle)	CFL1	2.8
F8W1K8	Ribosomal protein, large, P0	RPLP0	3.2
B4E022	Transketolase	TKT	3.2
P19338	Nucleolin	NCL	3.3
H0Y875	Calumenin	CALU	3.7
P22314	Ubiquitin-like modifier activating enzyme 1	UBA1	3.7
Q15365	Poly(rC) binding protein 1	PCBP1	3.9
F8W1N5	Nascent polypeptide-associated complex alpha subunit	NACA	4.2
P46940	IQ motif containing GTPase activating protein 1	IQGAP1	4.9
P23284	Peptidylprolyl isomerase B (cyclophilin B)	PPIB	5.1
P48643	Chaperonin containing TCP1, subunit 5 (epsilon)	CCT5	6.3
H3BT97	Matrix metallopeptidase 15 (membrane-inserted)	MMP15	7.1
B0QY90	Eukaryotic translation initiation factor 3, subunit L	EIF3L	8.5
B4DFK6	Calponin 3, acidic	CNN3	9.9
P09382	Lectin, galactoside-binding, soluble, 1	LGALS1	11.7
Q13492	Phosphatidylinositol binding clathrin assembly protein	PICALM	17.1
Q7KZF4	Staphylococcal nuclease and tudor domain containing 1	SND1	18.1
F6USW4	Capping protein (actin filament) muscle Z-line, beta	CAPZB	23
Q9Y4K1	Absent in melanoma 1	AIM1	24.2
P40227	T-complex protein 1 subunit zeta	CCT6A	32.3
H0YEN5	Ribosomal protein S2	RPS2	34.6
P05386	Ribosomal protein, large, P1	RPLP1	35
E9PPU1	Ribosomal protein S3	RPS3	35.4
P23246	Splicing factor proline/glutamine-rich	SFPQ	45.8
P26038	Moesin	MSN	46.9
Q96AG4	Leucine rich repeat containing 59	LRRC59	60.9
J3KR87	Clathrin heavy chain 2	CLTCL1	63.7
Q13813	Spectrin, alpha, non-erythrocytic 1	SPTAN1	65.9
C9J9K3	Ribosomal protein SA	RPSA	66.6
E9PH38	Protein phosphatase 2, regulatory subunit A, alpha	PPP2R1A	67.1
O95573	Acyl-CoA synthetase long-chain family member 3	ACSL3	68
E7EP94	Heat shock 70kDa protein 1A	HSPA1A	68
Q15056	Eukaryotic translation initiation factor 4H	EIF4H	84.6

To identify cellular pathways responsive to CuO NP exposure, we conducted Ingenuity Pathway Analysis (IPA) with 186 proteins identified by SILAC. The top five significant molecular functions were cell death and survival, protein synthesis, post-translational modification, protein folding, cellular development, and cellular growth and proliferation. The effects on protein synthesis and folding are consistent with the recent proteomics analysis of macrophage response to CuO NP [19], and could indicate reduced intracellular concentrations of proteins. Additional significant functions detected in our study included cell cycle, cell morphology, and cellular assembly and organization (Table 3). All of these functions are expected to be part of cellular response to CuO NP based on precedence in literature and thus validate our results. The top signaling pathway identified was actin cytoskeleton signaling, while the rest of the top five pathways identified included remodeling of epithelial adherens junctions, ILK signaling, epithelial adherens junction signaling and 14-3-3-mediated signaling pathways (Table 3). The significant metabolic pathways identified included glycolysis, gluconeogenesis and sucrose degradation.

**Table 3:** Top ten molecular functions and signaling pathways represented by proteins identified from BEAS-2B cells upon exposure for 24 h.

Molecular functions	Signaling Pathways
Cell Death and Survival	Actin Cytoskeleton Signaling
Protein Synthesis	Remodeling of Epithelial Adherens Junctions
Post-Translational Modification	ILK Signaling
Protein Folding	Epithelial Adherens Junction Signaling
Cellular Development	14-3-3-mediated Signaling
Cellular Growth and Proliferation	EIF2 Signaling
Cellular Movement	Germ Cell-Sertoli Cell Junction Signaling
Cell Cycle	RhoA Signaling
Cell Morphology	Regulation of Actin-based Motility by Rho
Cellular Assembly and Organization	Sertoli Cell-Sertoli Cell Junction Signaling

The reported lung epithelial cell response to CuO NP using DNA microarray and Gene Ontology (GO) analysis in A549 cells [18] was at a concentration of 25 µg/mL CuO NP (compared to the ∼0.09 µg/mL used here). This study discovered that the NP treatment resulted in down-regulation of proliferating cell nuclear antigen (PCNA), cell division control 2 (CDC2), cyclin B1 (CCNB1), target protein for Xklp2 (TPX2), and aurora kinase A (AURKA) and B (AURKB) and up-regulation of such proteins as nuclear receptors NR4A1 and NR4A3 and growth arrest and DNA damage-inducible 45 β and γ (GADD45B and GADD45G). None of the above-mentioned genes/proteins were identified as significantly regulated by our proteomics study, although some of the pathways altered by CuO NP were the same for both studies [29], such as Cellular Assembly and Organization identified by our pathway analysis and by Hanagata, Xu et al. [29].

Moreover, another published study focusing on the protein expression of mouse RAW 264.7 macrophage cells response to metallic copper and CuO NP [19]. Whole cell extracts were separated by 2D gels and several of the excised gel spots were identified by mass spectrometry, of which only seven proteins were found to be differentially expressed in response to CuO NP. Expression of ferritin light chain and L-lactate dehydrogenase were specific to Cu ions. Additional proteins such as glutamate-cysteine ligase regulatory subunit, 60S acidic ribosomal protein P0, peroxiredoxin-1 adenosylhomocysteinase and heme oxygenase 1 were seen as being affected by CuO NP as well. The overlap between the findings reported here with BEAS-2B cells and the published RAW 264.7 macrophage response to CuO NP is very limited. Only two proteins are common to both datasets and they are adenosylhomocysteinase and peroxiredoxin-2. This difference in cellular response could be attributed to the differences in the cell types evaluated as well as the CuO NP concentration and the size of NP used. Nevertheless, proteins identified as being responsive to CuO NP in our study and the published proteomic study still represent cellular processes such as oxidative stress, changes in cytoskeleton, protein metabolism etc., indicating that although the specific molecular players involved in a pathway could differ in different cells in response to CuO NP, there is a possible conservation at the higher order of cellular processes.

### Analysis of differentially expressed (DE) proteins identified by SILAC

The predominant molecular functions represented by the differentially expressed (DE) proteins (Table 4) were cellular function and maintenance, protein synthesis, cell death and survival, cell cycle and cellular movement. The DE proteins represented EIF2 signaling, regulation of EIF4 and p70S6K signaling, mTOR signaling, leukocyte extravasation signaling, remodeling of epithelial adherens junctions and actin cytoskeleton signaling (Table 4). The mammalian target of rapamycin mTOR activation results in the phosphorylation of a number of targets including a repressor of EIF4 and 4EBP1, which ultimately results in increased translation [30]. The mTOR protein is known to promote additional cellular processes such as cell growth and cell proliferation in response to growth factors including epidermal growth factor, nutrients and cellular stress [31], [32] of the mTOR signaling pathway indicates that CuO NP could elicit general cellular response to stress. Identification of two signaling pathways pertaining to protein translation, such as EIF2 and EIF4 signaling clearly indicates that exposure of CuO NP affects protein synthesis, possibly at the initiation step of translation. Effects on EIF3 (−6.2 fold EIF3F and 8.5 fold EIF3L) could impact the 43S pre-initiation complex and subsequent recruitment of mRNA to this activated pre-initiation complex by combined action of EIF3 and EIF4 (84 fold) [33].

**Table 4:** Top ten molecular functions and signaling pathways represented by BEAS-2B proteins with significant changes in expression upon CuO NP exposure for 24 h.

Molecular Functions	Signaling pathways
Cellular Function and Maintenance	IF2 Signaling
Protein Synthesis	Regulation of eIF4 and p70S6K Signaling
Cell Death and Survival	mTOR Signaling
Cell Cycle	Leukocyte Extravasation Signaling
Cellular Movement	Remodeling of Epithelial Adherens Junctions
Cellular Assembly and Organization	Actin Cytoskeleton Signaling
Cell Morphology	Aldosterone Signaling in Epithelial Cells
DNA Replication, Recombination, and Repair	Germ Cell-Sertoli Cell Junction Signaling
Lipid Metabolism	Epithelial Adherens Junction Signaling
Molecular Transport	Protein Ubiquitination Pathway

Increased expression of ribosomal phosphoproteins RPLP1 (35 fold), and RPS3 (35.4 fold), constituents of the eukaryotic translational machinery, could impact ribosome assembly as well as protein translation. RPS3 is involved in mRNA-aminoacyl tRNA interactions during protein synthesis. It can be cross-linked with translation initiation factor EIF-3 (EIF3L, 8.5 fold), known to be important for the initiation of protein synthesis. The accumulation of these proteins could be due to their induced expression, defective ribosome synthesis or post-translational regulation. Impaired ribosomal synthesis can trigger p53-mediated cell cycle arrest [34] and in such a scenario, extra ribosomal functions of ribosomal proteins could be important. RPS3 has multiple extra ribosomal functions. Like RPL23, RPS3 can activate p53 via the p53/MDM2 (mouse homolog of HDM2) regulatory loop by binding to both MDM2 and p53 [35] directly. This interaction protects p53 from MDM2 ubiquitination and degradation. Under conditions of oxidative stress and DNA damage, the MDM2/p53 complex is destabilized by phosphorylation of both proteins, resulting in the activation of p53. In addition to this destabilization, there is an enhanced interaction between p53 and RPS3. As a non-Rel homology protein member of the native NF-κB complex in the cytoplasm as well as the nucleus, RPS3 plays an important role in NF-kB mediated signaling and apoptosis. In response to DNA damage due to intrinsic/extrinsic stress, RPS3 associates with TRADD at the TNFR1 DISC and induces apoptosis in cells via the JNK pathway dependent on caspases. Of interest to this observation is the fact that RPS3 itself is a DNA repair enzyme. It can cleave apurinic/apyrimidinic (AP) DNA and has been shown to enhance the catalytic activity of base excision repair (BER) enzymes N-glycosylase/AP lyase [36]. Therefore, RPS3 is an important bridging factor between cellular damage, cell cycle arrest and apoptosis. This important mediator of multiple cellular processes is regulated by post-translational modifications such as phosphorylation, methylation and sumoylation. ERK-mediated phosphorylation of RPS3 in response to genomic damage is required for its translocation from the cytoplasm to the nucleus [37]. ERK-mediated Thr-42 phosphorylation of RPS3 prevents RPS3 integration with the ribosome. Protein phosphatase 2 dephosphorylates non-ribosomal RPS3 in the nucleus [38], enabling it to be assembled into the 40S ribosome. In response to CuO NP, expression of protein phosphatase 2, regulatory subunit A, increased (PPP2R1a, 67.1 fold). This increased expression could reduce the amount of RPS3 in the nucleus and negatively impact the aforementioned nuclear interactions of NF-kB and p53 critical for linking DNA damage to apoptosis. Dephosphorylation of RPS3 could sequester this protein to the ribosome, thus enhancing protein synthesis.

**Figure 5:**
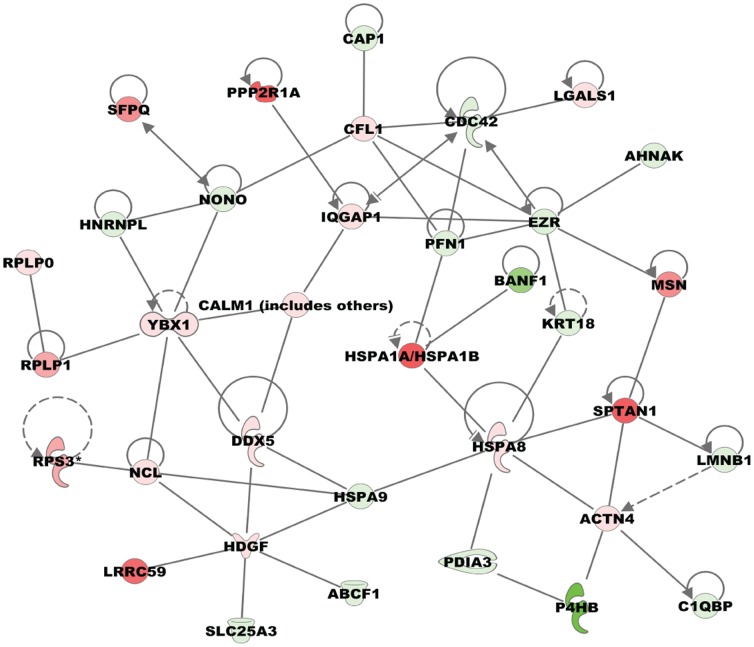
Top network identified by Ingenuity Pathways Analysis from BEAS-2B proteins upon CuO NP exposure. Ingenuity pathways analysis identified networks represented by BEAS-2B proteins differentially expressed upon a 24 h exposure to 0.01 µg/cm^2^ CuO NP. Associated molecular functions for the top network shown here include ‘Cell Cycle, Cellular Movement, Cellular Function and Maintenance’ molecular functions. In this network, the nodes represent proteins while the edges represent interaction between the proteins. Protein expression change in response to CuO NP is indicated by different colors (red represents increase; green denotes decrease in expression) with the intensity of the color corresponding to the magnitude of the fold change.

BEAS-2B cellular response to CuO NP exposure also affected multiple aspects of post-translational protein translocation via the endoplasmic reticulum (ER), including targeting, translocation and membrane insertion/secretion. Disruption of this process could result in accumulation of misfolded proteins marking ER stress and constitutes the unfolded protein response (UPR) pathway in cells; oxidative stress induced by the CuO NP could lead to the activation of the UPR pathway, which in turn can result in apoptosis [39]. Expression of nascent polypeptide-associated complex (NACA, 4.2 fold) was also increased. NACA is a protein that acts as a checkpoint protein to ensure that non-secretory/membrane proteins are not targeted to ER. Recognition of a nascent polypeptide signal sequence by a signal recognition particle (SRP) in the cytoplasm is followed by the tethering of SRP and the polypeptide complex to SSR as the first step of targeting. Reduced expression of ER chaperones and disulfide isomerases (P4HB, −88.8 fold; PDIA6, −1.7 fold) can interfere with proper folding of proteins leading to misfolding/aggregation. In response to this stress, expression of certain ER chaperones, like heat shock protein (HSPA8, 1.9 fold) and peptidyl prolyl isomerase B (PPIB, 5.1 fold) was increased, which could contribute to accelerated and/or accurate protein folding. In addition to its chaperone function in ER, reduced expression of mortalin (HSPA9, −12.1 fold) could affect protein folding in mitochondria. IPA identified a top molecular network that was centered on heat shock proteins indicating that a number of proteins that appear to be important for CuO NP mediated effects in BEAS-2B are indeed interacting with each other in the global protein network (Fig. 5). Although its exact function in ER stress is not clear, there is increased expression of a membrane-anchored protein (LRRC59, 61 fold) that localizes to the ER as well as the nuclear envelope. LRRC59 is required for the translocation of cytosolic fibroblast growth factor to the nucleus [40]. Some of the differentially expressed proteins were involved in post-transcriptional regulation of gene expression. Expression of RNA binding protein HNRNP L (−4.6 fold) was reduced. This multifunctional protein is involved in processing heterogeneous nuclear RNAs (hnRNAs) into mature mRNAs and also acts as a trans factor in regulating gene expression [41]. HNRNP L is known to affect the stability of VEGF RNA under conditions of hypoxia and it forms a stress response complex [42]. Expression of SFPQ (45.8 fold) increased upon CuO NP exposure. SFPQ is a splicing factor involved in alternative splicing of mRNA. Alternate splicing of as many as 50% of the multi-exon human genes generates proteomic diversity [37]. Therefore, the altered expression of proteins involved in this mechanism can have a major impact on gene regulation. Increased expression of nucleolin (NCL, 3.3 fold), a predominant nucleolar protein known to interact with histone H1 for chromatin decondensation, has implications in the overall transcriptional regulation of gene expression, specifically pre-RNA transcription and transcription elongation [43]. Expression of transgelin (TAGLN2, 1.6 fold), an actin binding protein involved in regulating androgen receptor (AR) function, increased in response to CuO NP exposure. In non-reproductive tissues, AR transcription factor controls the expression of genes involved in cell proliferation, cell growth, differentiation, and cell death [44]. Transgelin suppresses AR function by interrupting its heterodimerization with ARA54, which results in the retention of AR and ARA54 in the cytoplasm [45] and prevents AR nuclear function.

Acyl CoA synthetase (ACSL4, −97.4 fold) is an enzyme that esterifies arachidonic acid preferentially in cells. Unesterified arachidonic acid is an apoptotic signal [46], thus reduced expression of ACSL4 could also contribute to apoptotic mechanisms via caspase 3. Increased expression of αII spectrin (SPTAN1, 65.9 fold) in response to CuO NP exposure could constitute an epithelial cell stress response, as it can enhance the repair of DNA inter-strand cross-links. αII spectrin facilitates the recruitment of repair proteins. It is also important for maintaining chromosomal stability [47]. The SH3 domain of αII spectrin is involved in Rac activation and lamellipodia formation through its interactions with adhesion proteins.

### Validation experiments

#### Analysis of phosphorylated proteins by immunoblotting and mass spectrometry of IMAC enriched peptides

SILAC based proteomics identified a number of proteins that are involved in multiple signaling pathways. Signaling pathways are known to be regulated by post-translational modifications such as phosphorylation. Phosphorylation is one of the most abundant protein post-translational modifications regulating key molecular processes. In addition, we also detected differential expression of phosphorylation-regulating proteins like protein phosphatase 2 (PPP2R1A, 67.1 fold), which could impact phosphorylation sites of proteins in response to CuO NP. We hypothesized that CuO NP treatment altered phosphorylation of specific proteins and carried out the analysis of phosphorylated proteins.

**Figure 6:**
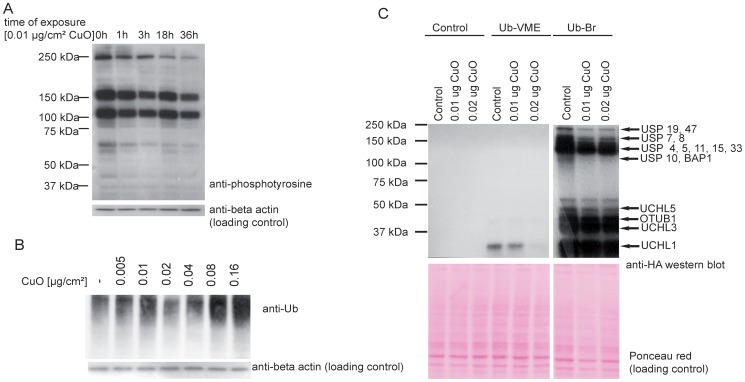
Effects of CuO NP on tyrosine phosphorylation and ubiquitination in human epithelial lung cells. (A). The CuO NP affects protein tyrosine phosphorylation. BEAS-2B cells were plated, left to recover for one day and exposed to the CuO NP (30–50 nm) at 0.01 µg/cm^2^ for the indicated times. The cells were lysed and equal amounts of protein were resolved by SDS-PAGE and subjected to anti-phosphotyrosine immunoblotting. Beta actin was used as a loading control. (B). The CuO NP affects bulk polyubiquitination in BEAS-2B cells. Cells were plated, left to recover for one day and exposed to the CuO NP (30–50 nm) at indicated concentrations for 18 h. The cells were lysed and equal amounts of protein were resolved by SDS-PAGE and subjected to anti-ubiquitin immunoblotting with beta actin as a loading control. (C). The CuO NP affects activity of deubiquitinases. BEAS-2B cells were plated, left to recover for one day and exposed to the CuO NP (30–50 nm) at 0.01 µg/cm^2^ and 0.02 µg/cm^2^ for 24 h. The cells were lysed and equal amounts of protein were incubated with ubiquitin-specific active-site probes (ubiquitin vinyl methyl ester, Ub-VME, and ubiquitin bromoaldehyde, Ub-Br, both HA-tagged) for 45 min at 37°C, or left without any treatment (Control). The reaction was resolved by SDS-PAGE and subjected to anti-HA immunoblotting to visualize active deubiquitinases, i.e. the ones that reacted with the HA-tagged probes. Estimated molecular weights (based on [66]) are indicated on the right.

We did an anti-phosphotyrosine western blot on protein samples from cells exposed to CuO NP for 24 h, which indicated that phosphorylation of several proteins is indeed regulated (Fig. 6A). Proteins isolated from BEAS-2B exposed to CuO NP for 24 h were enriched for phosphorylated proteins by using IMAC enrichment. This method is selective for serine/tyrosine/threonine phosphorylation of proteins. The IMAC enriched proteins were subjected to mass spectrometry-based quantitative proteomics. We identified 32 proteins with differentially regulated phosphopeptides in response to CuO NP treatment (Table 5), mostly phosphorylated on serine residues. Tyrosine phosphorylation is not as abundant as serine or threonine phosphorylation (the ratio of phosphorylation on phosphoserine:phosphothreonine:phosphotyrosine is 1,800∶200∶1 [48]), which explains its underrepresentation in phosphoproteomics data. Compared to untreated control sample, 18 proteins had reduced levels of phosphopeptides, i.e. these proteins are most likely to be de-phosphorylated or possibly less expressed. Based on IPA analysis, the molecular functions represented by CuO NP responsive phosphoproteins include protein synthesis, gene expression, post-translational modification, protein folding and cellular assembly and organization. The identified proteins are part of multiple signaling pathways including lipid antigen presentation by CD1, telomerase signaling, and protein ubiquitination pathways. All of these functions and pathways could be affected by dephosphorylation and/or phosphorylation.

**Table 5:** BEAS-2B proteins with significant changes in phosphorylation status in response to CuO NP exposure for 24 h.

Accession	Fold change	Symbol	Modifications	Sequence
K7EL60	−94.7	EIF3G	S10(Phospho)	GIPLATGDTsPEPELLPGAPLPPPK
Q9H1E3	−78.7	NUCKS1	S11(Phospho)	KVVDYSQFQEsDDADEDYGR
Q09666	−72.9	AHNAK	S9(Phospho)	LPSGSGAAsPTGSAVDIR
P46821	−56.5	MAP1B	S3(Phospho)	TTsPPEVSGYSYEK
Q9Y5Y9	−54.8	SCN10A	N-Term(Acetyl); S17(Phospho); S21(Phospho); T22(Phospho)	dESVPQVPAEGVDDTSsSEGstVDCLDPEEILR
P49736	−47.2	MCM2	S6(Phospho)	GLLYDsDEEDEERPAR
Q12906	−45.3	ILF3	S2(Phospho); K4(Label:13C(6)); K15(Label:13C(6)) K49(Label:13C(6))	DsSkGEDSAEETEAkPAVVAPAPVVEAVSTPSAAFPSDATAEQGPILTk
G5E9M5	−43.8	ILF3	S2(Phospho)	DsSKGEDSAEETEAKPAVVAPAPVVEAVSTPSAAFPSDATAENVK
P07355	−39.8	ANXA2	S12(Phospho)	LSLEGDHSTPPsAYGSVK
K7EMU2	−37.7	PRKAR1A	S5(Phospho)	EDEIsPPPPNPVVK
F8VS07	−29.1	LIMA1	S19(Phospho); K28(Label:13C(6))	EGHSLEMENENLVENGADsDEDDNSFLk
P55884	−28.5	EIF3B	S16(Phospho)	TEPAAEAEAASGPSEsPSPPAAEELPGSHAEPPVPAQGEAPGEQAR
O60841	−24.1	EIF5B	S9(Phospho)	NKPGPNIEsGNEDDDASFK
K7ESE3	−23.6	RAD23A	S4(Phospho)	EDKsPSEESAPTTSPESVSGSVPSSGSSGR
F5GXM1	−19.7	HDAC1	S22(Phospho); K32(Label:13C(6)); R33(Label:13C(6)15N(4))	MLPHAPGVQMQAIPEDAIPEEsGDEDEDDPDkr
H7BZ93	−16.2	SETD2	N-Term(Acetyl); T2(Phospho)	stLSKPIPKSDK
Q8NGP9	−4.6	OR5AR1	C5(Carbamidomethyl); T10(Phospho); T13(Phospho); Y16(Phospho); M20(Oxidation); Y23(Phospho)	AFSTcGSHLtGItLFyGTVmFMyLRPTSSYSLDQDK
Q96JM3	−1.6	CHAMP1	S6(Phospho); S16(Phospho)	LAPVPsPEPQKPAPVsPESVK
F8WE04	1.7	HSPB1	S3(Phospho); R10(Label:13C(6)15N(4))	QLsSGVSEIr
E9PS34	1.7	NAP1L4	S14(Phospho); K25(Label:13C(6))	EFITGDVEPTDAEsEWHSENEEEEk
P06748	1.8	NPM1	M11(Oxidation); S16(Phospho); K19(Label:13C(6))	DELHIVEAEAmNYEGsPIk
Q92538	2.8	GBF1	S10(Phospho)	ADAPDAGAQsDSELPSYHQNDVSLDR
H7C456	2.9	MAP4	S4(Phospho)	DMEsPTKLDVTLAK
Q96F25	4.0	ALG14	N-Term(Acetyl); T15(Phospho); M18(Oxidation)	kVIIVYVESICRVEtLSmSGK
H0YJU2	4.4	AHSA1	Y6(Phospho); T21(Phospho)	EAMGIyISTLKTGHFATITLtFIDK
H9KV91	7.4	AKT1S1	S18(Phospho); S22(Phospho)	AATAARPPAPPPAPQPPsPTPsPPRPTLAR
P61978	9.9	HNRNPK	S13(Phospho); C29(Carbamidomethyl)	IIPTLEEGLQLPsPTATSQLPLESDAVEcLNYQHYK
P06748	12.2	NPM1	C1(Carbamidomethyl); S22(Phospho)	cGSGPVHISGQHLVAVEEDAEsEDEEEEDVK
F8VRD4	13.6	RFX4	N-Term(Acetyl); T9(Phospho); S17(Phospho); T19(Phospho)	aISGVLMPtVLQALPDsLtQVIRKFAK
P46821	14.9	MAP1B	C23(Carbamidomethyl); S25(Phospho); K29(Label:13C(6))	VSAEAEVAPVSPEVTQEVVEEHcAsPEDk
Q9H5L6	49.5	THAP9	T16(Phospho); S19(Phospho); K24(Label:13C(6))	LSDIGITVLAVTSDAtAHsVQMAk
K7EL60	51.3	EIF3G	T9(Phospho); K25(Label:13C(6))	GIPLATGDtSPEPELLPGAPLPPPk

Subunits of eukaryotic translation initiation factor (EIF) 3 critical for the formation of the pre-initiation complex during the initial stages of translation were identified as being phosphorylated in response to CuO NP. Ser-83 phosphorylation of EIF3B was reduced (−28.5 fold). While the Ser-42 phosphorylated form of EIF3G was down regulated (−94.7 fold), there was an increase in Thr-41 phosphorylation (51.3 fold) of the same sub-unit. EIF3G is known to interact with cap dependent translation initiation factor and facilitate the recruitment of ribosomes in translation initiation that is cap dependent [49]. EIF3G was shown to be the target in apoptosis-inducing factor (AIF) mediated inhibition of protein synthesis [50]. Ser-214 phosphorylation of EIF5B (−24.1 fold) decreased in response to CuO NP. EIF5B, a GTPase that is involved in ribosomal sub-unit joining, has been shown to be critical for the stability of the 80S ribosome during translation [45]. A number of large scale approaches identified phosphorylation of EIF described above. In one study, phosphopeptides were enriched from the human HeLa cell line to identify mitotic phosphorylation by mass spectrometry. EIF3B, EIF3G and EIF5B [46] were identified as being phosphorylated, indicating that these proteins could be targets of cell cycle-specific kinases.

Thanatos-associated protein (THAP) domain is a Zinc finger motif with sequence-specific DNA-binding activity and this evolutionarily conserved motif has been identified in 12 human proteins (THAP 0–11) [51]. This protein family has been implicated in cell proliferation, regulation of transcription, and apoptosis [52]–[54]. THAP proteins also have HCF-1 binding motif, which facilitates their interaction with other DNA-binding factors that have histone modifying activities [48]. Thus THAP activity is postulated to be DNA and chromatin dependent. Recent evidence indicates that human THAP9 is related to *Drosophila* P-element transposase and has been shown to retain the catalytic activity capable of mobilizing P-transposable activity [55]. In this study, we identified that phosphorylation of Thr-376 and Ser-379 of THAP9 increased 49.5 fold in response to CuO NP exposure. Both of these modifications are outside the annotated Zinc finger (1–89) and HCF1-binding motif (123–126) in the protein. To our knowledge, this is the first report of a PTM for THAP9 protein.

Nucleophosmin (NPM1B23) is a ubiquitous chaperone phosphoprotein with multiple functions. As a histone chaperone, it is known to mediate chromatin assembly and disassembly. It is also involved in ribosome biogenesis and transport, has anti-apoptotic activity and regulates centrosome duplication and tumor suppressor genes such as p53 and ARF [52]. Expression of nucleophosmin has been shown to be required for proliferation of lung cancer cells [53]. NPM1 is phosphorylated by cyclin E/cdk2 during the G1 phase and threonine phosphorylated NPM1 dissociates from the centrosome, initiating its duplication. We identified up-regulation of Ser-70 (1.8 fold) and Ser-125 (12.2 fold) phosphorylation of this shuttle protein. Of these modifications, Ser-125 phosphorylation by Cdk2 was identified by phosphoproteomics [56]. Phosphorylation of NPM1 serine by polo-like kinase 1 is necessary for mitosis to be defect-free. Serine phosphorylation is important for the partitioning of NPM1 between the nucleoplasm and nucleolus and has implications for ribosome biogenesis and maintenance of the nucleolar structure. We identified an increase in Ser-121 phosphorylation for nucleosome assembly protein 1-like 4 (NAP1L4, 1.7 fold), which belongs to the nucleosome assembly protein (NAP) family. NAPs are histone chaperones that shuttle histones between the cytoplasm and nucleus. Translocation of these proteins is known to be regulated by serine phosphorylation, as described for NAP1 [57]. In this study, for the first time, we report phosphorylation of NAP1L14.

Phosphorylation of AKT1 substrate 1 on residues Ser-88 and Ser-92 (AKTS1, 7.4 fold) increased in response to CuO NP. AKTS1 is a negative regulator of mTOR kinase activity. AKT-mediated threonine phosphorylation and mTOR-mediated serine phosphorylation of AKTS1 relieves inhibition of mTOR [58]. The mTOR is a key regulator of cell growth and survival in response to growth hormone signals, nutrients and ATP levels. The increased levels of ATP due to increased expression of ATP synthase could be the trigger for the activation of the mTOR signaling pathway. The mTOR phosphorylates key translational and ribosome synthesis regulators and is known to reduce apoptosis [59]. Activation of mTOR by its phosphorylation could constitute the cellular response to counter cell death induced by CuO NP.

A protein involved in microtubule polymerization, microtubule-associated protein 1B (MAP1B, −56.5 fold) was also identified as being predominantly dephosphorylated on residue Ser-1965 upon CuO NP exposure. Serine phosphorylation of MAP1B by GSK3β kinase results in the loss of stable microtubules in COS-7 cells. Thus CuO NP appears to influence microtubule dynamics in growth cones [60]. Dephosphorylation of serine residues in AHNAK nucleoprotein, a ubiquitous phosphoprotein, can impact its localization. In epithelial cells, AHNAK is excluded from the nucleus upon serine phosphorylation by protein kinase B [59]. We identified dephosphorylation of Ser-216 of AHNAK (−72.9 fold) upon CuO NP exposure. In epithelial cells, depending on Ca2+ concentration, AHNAK is localized either in the cytosol or the plasma membrane. AHNAK interacts with protein kinase C-α (PKC-α). The PKC family of proteins plays an important role in the regulation of cellular functions such as cell division, survival, and proliferation through the Erk/MAPK cascade [61]. Another nuclear protein that was identified as having reduced phosphorylation (Ser-19, 78.7 fold) upon CuO NP exposure is nuclear casein kinase and cyclin-dependent kinase substrate (NUCKS1). This highly conserved protein contains several consensus serine/threonine phosphorylation sites for casein kinase II and cyclin-dependent kinases. While it is known that NUCKS1 is phosphorylated *in vivo* by Cdk1 during mitosis of the cell cycle, the effect of this phosphorylation is not clear [62].

CuO NP also affected a protein involved in chromatin remodeling and thereby transcriptional gene regulation. Ser-200 phosphorylation of histone deacetylase 1 (HDAC1, −19.7 fold) was significantly reduced. Dephosphorylated HDAC cannot bind core histones efficiently for deacetylation, which leads to chromatin condensation [63].

#### Labeling of protein lysates with ubiquitin-specific active-site probes

Protein ubiquitination was identified amongst the top ten functions represented by differentially expressed proteins (Table 4). Furthermore, this pathway has been one of only three significant pathways represented in our phosphoproteomics data. Thus our data suggests that CuO NP induce changes in the ubiquitin proteasome system. Protein ubiquitination has been already shown to play major functions in apoptosis [64]. To validate this hypothesis, we analyzed levels of polyubiquitinated proteins in cells treated or not treated with CuO NP for 24 h (Fig. 6B) by using anti-ubiquitin western blotting. We observed an increased amount of polyubiquitination in CuO NP treated cells. This could be due to impaired activity of the 26S proteasome, its associated proteins or changes in activity of ubiquitin-specific proteases, or alternatively, is caused by the induction of polyubiquitination due to the accumulation of misfolded proteins. We identified unfolded protein response as one of the molecular functions represented by the differentially expressed proteins. Since deubiquitinases are known to play important functions in regulation of polyubiquitination status of protein substrates [65], we determined whether the activity and/or protein levels of any deubiquitinases was affected in cells after the CuO NP treatment. We utilized ubiquitin-specific active-site probe labeling to visualize active deubiquitinases (Fig. 6C). For the UB-VME-HA probe labeling, we included results that clearly showed differential labeling of catalytically active UCH-L1, although longer exposures indicated labeling of other DUBs as well. Several deubiquitinases, including UCH-L1, are clearly shown to be down-regulated after CuO NP treatment. Future studies are needed to identify the specific deubiquitinases that are regulated by CuO NP, since these could potentially play important roles in cell stress induced by CuO NP. Based on estimated molecular weight [66], one possible deubiquitinase that could be regulated is USP47, and we have already shown USP47 to be important in DNA repair as it is required for stability of newly synthesized cytoplasmic Pol β [67].

#### Analysis of actin cytoskeleton

**Figure 7:**
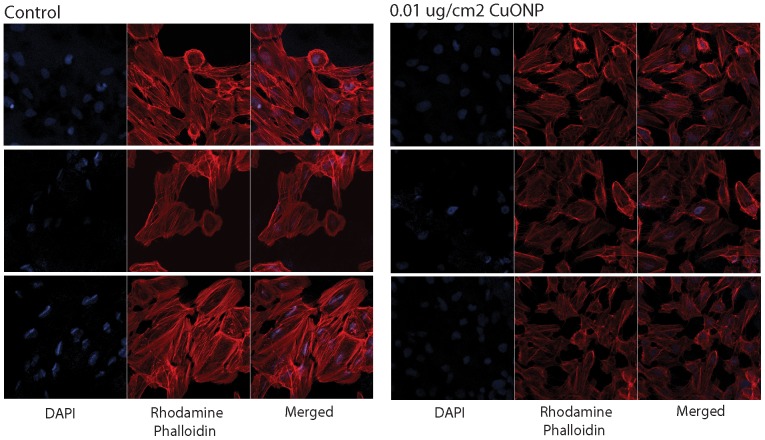
CuO NP affects actin cytoskeleton in BEAS-2B cells. BEAS-2B cells were exposed or not to 0.01 ug/cm^2^ CuO NP for 24 h and stained by using rhodamine phalloidin to visualize filamentous actin and with DAPI to visualize nucleus. The specimens were analyzed by confocal microscopy. The gain and exposure times were kept constant for both of the samples and the DAPI staining was used as a control. The analysis of triplicate samples suggested that the CuO NP-treated cells have reduced number of stress fibers and diminished overall amount of F-actin.

Actin cytoskeleton signaling was the top signaling pathway represented in the identified proteome (Table 3), cellular movement is one of the functions associated with the top network (Fig. 5) identified from the differentially expressed proteins, and actin cytoskeleton signaling was also significant in the differentially expressed proteins (Table 4), although expression of actin itself was not affected. If indeed CuO NP exposure results in the alteration of the actin cytoskeleton signaling pathway, this could affect the structure of the actin cytoskeleton in BEAS-2B cells. We tested for this expected phenotype by immunostaining and visualization of F-actin (as opposed to G-actin) by using confocal microscopy. We stained BEAS-2B cells exposed to CuO NP for 24 h with rhodamine phalloidin to visualize filamentous actin and with DAPI to visualize nuclei by confocal microscopy (Fig. 7). Our results indicate that CuO NP-treated cells have a reduced number of stress fibers and diminished overall amount of filamentous actin (F-actin), as compared to control cells that have F-actin in the cell periphery and stress fibers. Interestingly, it has previously been shown that 13 nm-diameter silver NP internalized by human dermal fibroblasts also reduced the amount of actin stress fibers in these cells [63]. Since organization of F-actin stress fibers is necessary for proper function of cells, cells with disrupted F-actin cytoskeleton might have altered function, i.e. they are not attaching properly to the extracellular matrix [64] and might have detrimental effects on cells.

## Conclusions

Although the toxic effects of nanoparticles on epithelial cells have been documented, there is still a need to identify specific molecular mechanisms and protein pathways affected by CuO NP in cells. Our proteomics study was an attempt to quantify proteins and phosphoproteins that are down- or up-regulated in BEAS-2B lung epithelial cells exposed to CuO NP at a concentration of 0.01 µg/cm^2^. The Ingenuity Pathway Analysis Software was the used to help us interpret this data. This revealed that CuO NP had effects on protein homeostasis and negatively affected protein synthesis specifically by targeting eukaryotic translation initiation factors, such as EIF3 involved in pre-initiation complex formation as well as proteins like EIF5B that are required for stabilization of the 80S complex. CuO NP mediated effects on protein synthesis could be due to the altered phosphorylation state of translation initiation factors as identified by phosphoproteomics. A specific mechanism of cell death that we identified by proteomics was via the activation of androgen receptor mediated activation of genes involved in cell proliferation and death. Our proteomics data and follow up validation experiments also demonstrate that actin cytoskeleton and protein ubiquitination are disturbed in cells treated with nanoparticles. All of these results provide novel insights into the pathways affected by CuO NP, which could prove to be useful in designing future pharmacological interventions to counteract the harmful effects of this nanoparticle.

## Supporting Information

Information S1 **Supporting Tables S1-S4.**
(XLSX)
